# Therapeutic treatment with an oral prodrug of the remdesivir parental nucleoside is protective against SARS-CoV-2 pathogenesis in mice

**DOI:** 10.1126/scitranslmed.abm3410

**Published:** 2022-03-22

**Authors:** Alexandra Schäfer, David R. Martinez, John J. Won, Rita M. Meganck, Fernando R. Moreira, Ariane J. Brown, Kendra L. Gully, Mark R. Zweigart, William S. Conrad, Samantha R. May, Stephanie Dong, Rao Kalla, Kwon Chun, Venice Du Pont, Darius Babusis, Jennifer Tang, Eisuke Murakami, Raju Subramanian, Kimberly T. Barrett, Blake J. Bleier, Roy Bannister, Joy Y. Feng, John P. Bilello, Tomas Cihlar, Richard L. Mackman, Stephanie A. Montgomery, Ralph S. Baric, Timothy P. Sheahan

**Affiliations:** ^1^ Department of Epidemiology, University of North Carolina at Chapel Hill, Chapel Hill, NC, 27599, USA; ^2^ Gilead Sciences, Inc, Foster City, CA, 94404, USA; ^3^ Department of Pathology and Laboratory Medicine, University of North Carolina School of Medicine, Chapel Hill, NC, 27599, USA; ^4^ Lineberger Comprehensive Cancer Center, University of North Carolina School of Medicine, Chapel Hill, NC, 27599, USA.

## Abstract

The coronavirus disease 2019 (COVID-19) pandemic remains uncontrolled despite the rapid rollout of safe and effective severe acute respiratory syndrome coronavirus 2 (SARS-CoV-2) vaccines, underscoring the need to develop highly effective antivirals. In the setting of waning immunity from infection and vaccination, breakthrough infections are becoming increasingly common and treatment options remain limited. Additionally, the emergence of SARS-CoV-2 variants of concern, with their potential to escape neutralization by therapeutic monoclonal antibodies, emphasizes the need to develop second-generation oral antivirals targeting highly conserved viral proteins that can be rapidly deployed to outpatients. Here, we demonstrate the in vitro antiviral activity and in vivo therapeutic efficacy of GS-621763, an orally bioavailable prodrug of GS-441524, the parent nucleoside of remdesivir, which targets the highly conserved virus RNA-dependent RNA polymerase. GS-621763 exhibited antiviral activity against SARS-CoV-2 in lung cell lines and two different human primary lung cell culture systems. GS-621763 was also potently antiviral against a genetically unrelated emerging coronavirus, Middle East Respiratory Syndrome CoV (MERS-CoV). The dose-proportional pharmacokinetic profile observed after oral administration of GS-621763 translated to dose-dependent antiviral activity in mice infected with SARS-CoV-2. Therapeutic GS-621763 administration reduced viral load and lung pathology; treatment also improved pulmonary function in COVID-19 mouse model. A direct comparison of GS-621763 with molnupiravir, an oral nucleoside analog antiviral which has recently received EUA approval, proved both drugs to be similarly efficacious in mice. These data support the exploration of GS-441524 oral prodrugs for the treatment of COVID-19.

## Teaser

The oral prodrug, GS-621763, is potently antiviral against SARS-CoV-2 in vitro and in vivo.

## INTRODUCTION

Severe acute respiratory syndrome coronavirus 2 (SARS-CoV-2) emerged in December 2019 and has caused over 450 million infections and over 6 million deaths worldwide as of March 2022 (*1*–*3*). Although there are multiple effective vaccines, vaccination rates have lagged in the United States due to vaccine hesitancy and public mistrust, thus delaying the generation of population-level immunity required to diminish community spread. In addition, outside of the United States, many countries do not have equitable access to vaccines or have been slow to vaccinate (*4*–*7*). This collection of events has fueled the generation of viral variants that are increasingly transmissible and more capable of escaping human immunity. Therefore, there is an immediate unmet need for oral antivirals that can be rapidly disseminated to treat coronavirus disease 2019 (COVID-19). Next-generation oral coronavirus (CoV) antivirals, if widely disseminated and given early in infection, could curtail the duration of disease, reduce long-term sequelae of COVID-19, minimize household transmissions, and lessen hospitalizations, thus having a broad impact on public health.

There are multiple direct-acting antiviral (DAA) therapies in use to treat COVID-19 (*8*–*12*), including Emergency Use Authorization (EUA)-approved monoclonal antibodies (mAbs) and FDA-approved remdesivir (RDV, GS-5734). Monoclonal antibodies have demonstrated efficacy for treating active COVID-19 cases in outpatients but currently all mAbs must be administered through injection, limiting their use to those with ready access to healthcare (*13*–*15*). In addition, several SARS-CoV-2 variants of concern (VOCs) have evolved that are resistant to first-line mAb therapies (*16*, *17*). Currently, RDV is the only FDA-approved small-molecule direct-acting antiviral to treat COVID-19, but there are several other DAAs that are currently in human clinical trials or that have received EUA approval, including nucleoside analog molnupiravir (MPV, EIDD-2801), as well as the MPro inhibitor, Paxlovid (PF-07321332) (*18*–*26*). Unlike mAbs, which specifically target the virion surface exposed spike protein of SARS-CoV-2, nucleoside analog drugs target a viral enzyme that is highly conserved among CoV, the RNA-dependent RNA polymerase (RdRp) nsp12; this renders them broadly active against multiple emerging, endemic, and enzootic CoV. Moreover, due to its high degree of conservation among CoV, the RdRp likely does not have the same capacity for mutational change as spike, which may translate into RdRp having a higher barrier to resistance (*19*–*21*).

Despite demonstrated therapeutic efficacy of RDV against SARS-CoV-2 in animal models (*18*, *24*, *27*) and in human clinical trials (*8*), the requirement of intravenous administration has limited its widespread use during this pandemic. In contrast, the orally bioavailable nucleoside prodrug GS-621763, is designed for optimal delivery of the parent nucleoside GS-441524 into systemic circulation, which is then metabolized inside cells into the same active nucleoside triphosphate formed by RDV (*28*). Here, we detail the in vitro antiviral activity in various cell models and in vivo therapeutic efficacy of oral GS-621763 in a mouse model of SARS-CoV-2 pathogenesis.

## RESULTS

### GS-621763 has antiviral activity against SARS-CoV-2 in cell lines and human primary cell cultures.

**Fig. 1.**
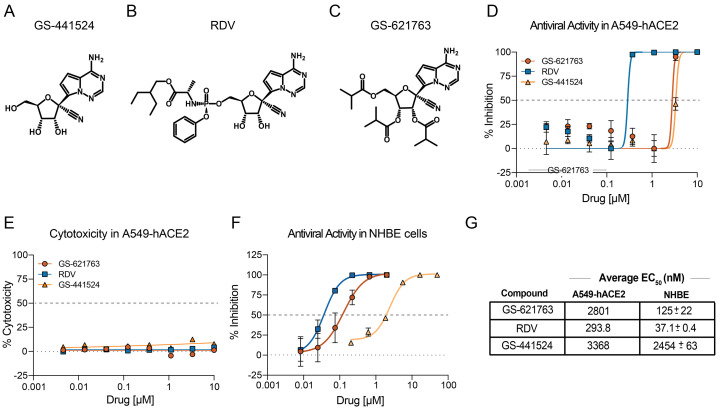
The chemical structure and in vitro potency of GS-621763 in comparison to RDV (GS-5734) and GS-441524. (**A to C**) The chemical structures of the parent adenosine nucleoside analog GS-441524 (A), the monophosphoramidate prodrug RDV (B), and GS-621763, the tri-isobutyryl ester of GS-441524 (C) are shown. (**D**) The mean percent inhibition of SARS-CoV-2 replication by GS-621763, in comparison to the prodrug RDV and the parental nucleoside GS-441524 in A459-hACE2 cells is shown (triplicate samples were analyzed). (**E**) Cytotoxicity was measured in A459-hACE2 cells treated with GS-621763, RDV, and GS-441524 (triplicate samples were analyzed). (**F**) Inhibition of SARS-CoV-2-fLUC replication by GS-621763, RDV, and the parental nucleoside GS-441524 in normal human bronchial epithelial (NHBE) cultures is shown (duplicate samples were analyzed). (**G**) In vitro EC_50_ values are shown for inhibition of viral replication by GS-621763, RDV, and the parental nucleoside GS-441524 in A459-hACE2 and NHBE cells. Data are presented as mean ± SD. For (D to F), the horizontal dashed line indicates 50% inhibition on the X-axis and the dotted line indicates 0% inhibition on the X-axis.

GS-441524 is the parental adenosine nucleoside analog (Fig. 1A) of both the monophosphoramidate prodrug RDV (GS-5734, Fig. 1B), and the triester prodrug GS-621763 (Fig. 1C). All three molecules are metabolized to the same active nucleoside triphosphate in cells, but through different activation pathways. GS-621763 is rapidly metabolized during oral absorption to GS-441524, then intracellularly converted by cellular kinases to the analog monophosphate metabolite before further metabolism to the active nucleoside triphosphate. In contrast, the intact phosphoramidate prodrug, RDV, is broken down inside cells directly to the same monophosphate metabolite, effectively bypassing the rate-limiting first phosphorylation step of GS-441524 (*28*). To determine if GS-621763 could inhibit replication of SARS-CoV-2 in cellular assays, we first evaluated its antiviral activity against a SARS-CoV-2 reporter virus expressing nanoluciferase (SARS-CoV-2 nLUC) in A549-hACE2 cells stably expressing the human entry receptor angiotensin-converting enzyme 2 (ACE2) (*29*). With GS-621763, we observed a dose-dependent antiviral effect on SARS-CoV-2 nLUC replication with an average half-maximum effective concentration (EC_50_) of 2.8 μM (Fig. 1D, and fig. S1A). In the same assay, we measured EC_50_ values for the control compound RDV of 0.29 μM; this value is similar to those reported previously in these cells and is reflective of the enhanced ability of the phosphoramidate prodrug to rapidly and efficiently generate active triphosphate by bypassing the slower initial phosphorylation step (Fig. 1D, fig. S1A) (*30*). As was observed in other cell systems, the parental nucleoside, GS-441524, was less potent (EC_50_ = 3.4 μM) than RDV in our assay and was similar in potency to GS-621763. This suggests that the tri-isobutyryl esters of GS-621763 are efficiently cleaved in the assay to release GS-441524 (Fig. 1D, fig. S1A). Importantly, we did not observe any measurable cytotoxicity of any of the inhibitors in A549-hACE2 cells at concentrations up to 10 μM (Fig. 1E, fig. S1B). Antiviral assays in human primary lung cell systems provide insight into drug candidate uptake, metabolism, and antiviral activity in cells similar to those targeted by emerging CoV in humans. We first evaluated drug performance in normal human bronchial epithelial (NHBE) cultures infected with a recombinant SARS-CoV-2 expressing Firefly luciferase (SARS-CoV-2 fLUC). GS-621763, RDV, and GS-441524 inhibited SARS-CoV-2 replication in NHBE cultures with EC_50_ values of 0.125, 0.0371, and 2.454 μΜ, respectively (Fig. 1F, fig. S1C). The in vitro potency for all three compounds in A549-hACE2 and NHBE is summarized in Fig. 1G. Human primary airway epithelial (HAE) cell cultures model the cellular complexity and architecture of the human conducting airway (*19*). In HAE cell cultures infected with wild-type SARS-CoV-2 (*29*), we confirmed the antiviral activity of GS-621763 in a primary lung cell system complementary to NHBE (fig. S2). To gain insight into the breadth of antiviral activity of GS-621763 against non-SARS-related CoVs, we performed an antiviral assay with the betacoronavirus group 2C CoV, Middle East Respiratory Syndrome CoV (MERS-CoV), whose RdRp is 88% similar to that of SARS-CoV-2 (*19*). GS-621763 was potently antiviral (EC_50_ = 0.74 μM) against MERS-CoV in human lung epithelial cells (Calu-3 2B4) (fig. S3A and B). Paralleling SARS-CoV-2 data above, GS-621763 was more potent than GS-441524 (EC_50_ = 2.1 μM) but less potent than RDV (EC_50_ = 0.16 μM) (fig. S3A and B). All three compounds were not cytotoxic (50% cytotoxic concentration (CC_50_) > 10 μM) (fig. S3C). All together, these data show that GS-621763 is transported, metabolized, and potently antiviral in human primary cell systems that model the tissues targeted by SARS-CoV-2 in humans, and exerts antiviral activity against genetically divergent beta-CoVs.

### Therapeutic efficacy of GS-621763 is dose-dependent in mouse models of SARS-CoV-2 infection.

**Fig. 2.**
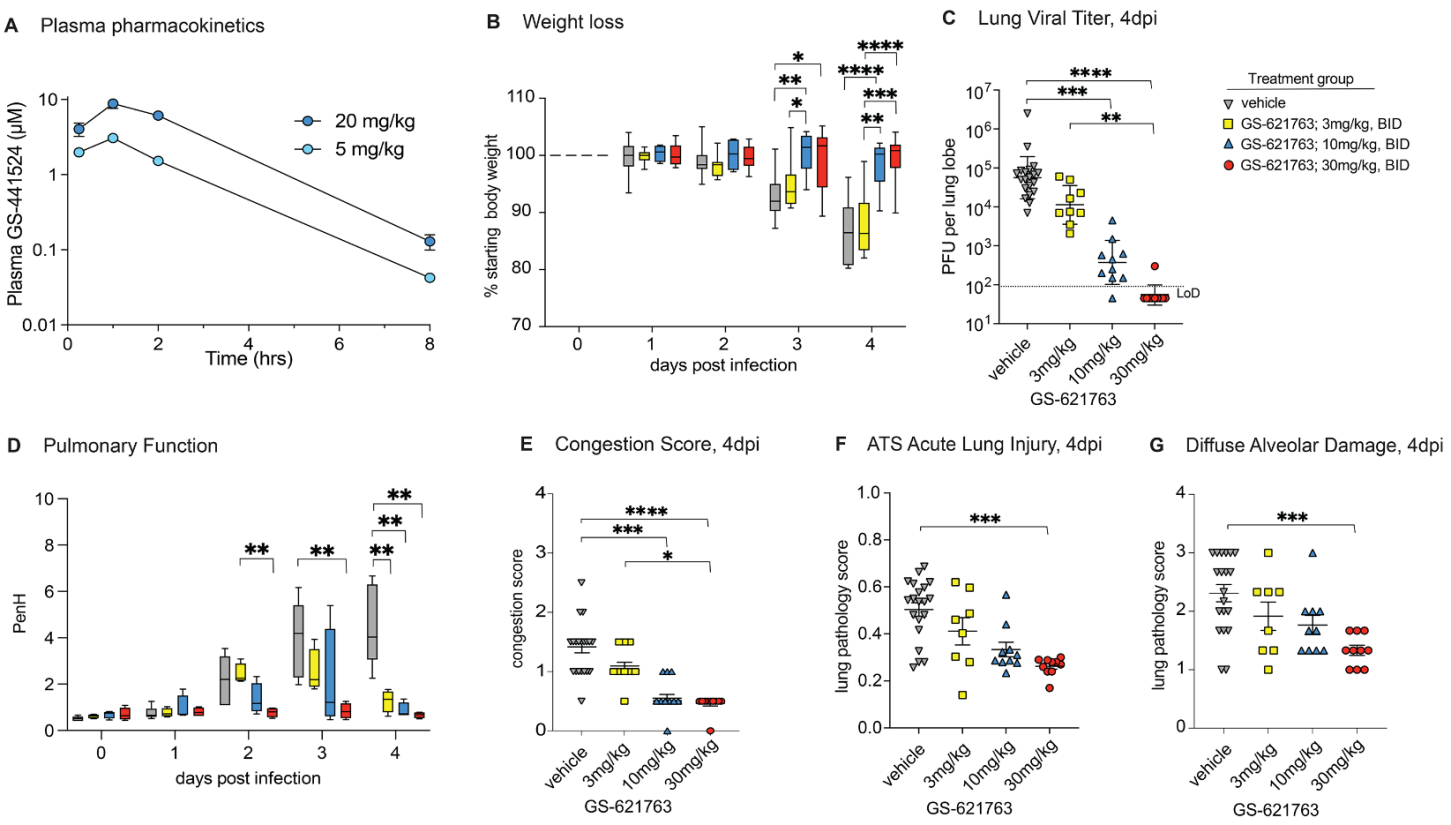
GS-621763 confers therapeutic protection against SARS-CoV-2 infection in mice in a dose-dependent manner. (**A**) Plasma pharmacokinetics of GS-441524 is shown in uninfected BALB/c mice following a single oral administration of GS-621763 at either 5 or 20 mg/kg. Plasma concentrations of GS-621763 were below the limit of quantification at all time points. (**B**) Depicted is the percent starting weight in mice treated therapeutically with vehicle (n=19) or 3 mg/kg (n=9), 10 mg/kg (n=10), and 30 mg/kg (n=10) GS-621763 at 8 hpi with 1x10^4^ PFU SARS-CoV-2 MA10. (**C**) Lung viral titers were measured in mice that were therapeutically treated with vehicle (n=19) or 3 mg/kg (n=9), 10 mg/kg (n=10), and 30 mg/kg (n=10) GS-621763 at 8 hpi with 1x10^4^ PFU SARS-CoV-2 MA10. The dashed line indicates the limit of detection (LoD). (**D**) Pulmonary function was measured in mice therapeutically treated with vehicle (n=7) or 3 mg/kg (n=4), 10 mg/kg (n=4), and 30 mg/kg (n=4) GS-621763 at 8 hpi with 1x10^4^ PFU SARS-CoV-2 MA10. PenH is a surrogate measure for bronchoconstriction. (**E**) Lung congestion scores were calculated in mice therapeutically treated with vehicle (n=19) or 3 mg/kg (n=9), 10 mg/kg (n=10), and 30 mg/kg (n=10) GS-621763 at 8 hpi with 1x10^4^ PFU SARS-CoV-2 MA10. (**F and G**) Lung pathology was quantified in mice therapeutically treated with vehicle (n=19) or 3 mg/kg (n=9), 10 mg/kg (n=10), and 30 mg/kg (n=10) GS-621763 at 8 hpi with 1x10^4^ PFU SARS-CoV-2 MA10. ATS acute lung injury (F) and diffuse alveolar damage (G) were scored in a blinded manner. Data were analyzed using a two-way ANOVA (weight loss and lung function) or a Kruskal-Wallis test (lung titer, congestion score, and pathology scores), *p<0.05, **p<0.005, ***p<0.0005, ****p<0.0001. Box and whisker plots in (B) and (D) show Minimum (Min) to Maximum (Max) values. Data in (C), (E), (F), and (G) are shown as mean ± SEM.

We have previously performed multiple studies describing the therapeutic efficacy of subcutaneously administered RDV in mice (*Ces1c* ^−/−^ C57BL/6J) genetically deleted for a secreted plasma carboxylesterase 1c (*Ces1c*) absent in humans that dramatically reduces drug half-life in wild-type mice (*18*–*20*, *27*, *31*). However, the prodrug GS-621763 is designed to be rapidly cleaved pre-systemically in vivo to release GS-441524 into circulation, with no or very minimal intact ester observed in plasma as has been observed in African green monkeys and ferrets (*28*, *32*). Therefore, GS-621763 can be studied in wild-type mice where it should also be rapidly converted to parent GS-441524. Plasma pharmacokinetics following a single oral administration of GS-621763 at either 5 or 20 mg/kg were first determined in uninfected BALB/c mice (Fig. 2A). Doses were selected to provide high plasma exposures of GS-441524 that would support active triphosphate formation in the lung and to confirm pharmacokinetic dose proportionality needed to project exposures in efficacy studies. Previous studies had shown that the parent nucleoside was at least 10-fold less efficient at generating lung triphosphate than RDV, on a molar basis, thus requiring higher plasma exposures of parental GS-441524 to account for the reduced metabolic efficiency (*28*). Intact ester prodrug, within the limit of quantitation (LOQ; 0.002 μM for GS-621763), was not observed in mice at any sampled time point. GS-441524 was both rapidly absorbed and then cleared from systemic circulation, exhibiting a short plasma half-life of approximately 1 hour and dose proportional increases in both maximal plasma concentrations. To better understand the pharmacokinetic and pharmacodynamic relationship for GS-621763, we performed a series of dose-finding studies in BALB/c mice infected with mouse-adapted SARS-CoV-2 (SARS-CoV-2 MA10) (*31*). In young adult BALB/c mice infected with 10^4^ plaque forming units (PFU) SARS-CoV-2 MA10, virus replicates to high titers in the respiratory tract, mice lose 15 to 20% of their body weight by 4 days post-infection (dpi), and acute lung injury and loss of pulmonary function is typically observed after virus replication peaks on 2 dpi (*31*). We first defined the minimum dosage sufficient for maximal therapeutic efficacy in BALB/c mice initiating twice daily (bis in die, BID) oral treatment with either vehicle control or 3 mg/kg, 10 mg/kg, or 30 mg/kg GS-621763 beginning 8 hours post infection (hpi) with 10^4^ PFU SARS-CoV-2 MA10 (Fig. 2B to G). Unlike vehicle or 3 mg/kg GS-621763 treated animals, mice receiving either 10 or 30 mg/kg GS-621763 were completely protected from weight loss, thus demonstrating that early oral antiviral therapy can prevent the progression of disease (Fig. 2B). Congruent with the weight loss phenotype, both 10 and 30 mg/kg GS-621763 treated animals had significantly reduced viral lung titers as compared to both the vehicle and 3 mg/kg treated groups (p=0.0001 and p<0.0001, respectively, Fig. 2C). To monitor the effect of drug treatment on pulmonary function, we performed daily whole-body plethysmography (WBP) with a subset of mice from each group (N = 4 per treatment group). The WBP metric PenH is a surrogate marker of bronchoconstriction or airway obstruction and has recently been shown to be elevated following emerging CoV infection (*19*). Unlike vehicle treated animals who exhibited a uniform increase in PenH scores over time, we observed a drug dose-dependent reduction in PenH with maximal improvement seen in the 30 mg/kg GS-621763 dose group (Fig. 2D). Mice treated with 3 and 10 mg/kg GS-621763 had impaired lung function at days 2 and 3 post infection, but lung function returned to baseline by 4 dpi for all GS-621763 treated animals (Fig. 2D). Consistent with weight loss, virus titer, and pulmonary function data, mice treated with 10 or 30 mg/kg had significantly reduced lung congestion, a gross pathologic feature characteristic of severe lung damage (p=0.0006 and p<0.0001, respectively, Fig. 2E). We then scored lung tissue sections for the histologic features of acute lung injury (ALI) using two complementary semiquantitative tools. First, using an ALI scoring tool created by the American Thoracic Society (ATS), we blindly evaluated three diseased fields per lung section for several features of ALI including alveolar septal thickening, neutrophils in the interstitium and in air spaces, proteinaceous debris in airspaces, and the presence of hyaline membranes. Only mice treated with 30 mg/kg had significantly reduced ALI scores (p=0.0002, Fig. 2F). Second, we used a complementary tool measuring the pathologic hallmark of ALI, diffuse alveolar damage (DAD). Mice receiving 30 mg/kg had significantly decreased DAD in their lungs (p=0.0010, Fig. 2G). Together, these data demonstrate that the oral delivery of the nucleoside analog GS-621763 can diminish SARS-CoV-2 virus replication and associated pulmonary disease in a dose-dependent manner.

### GS-621763 treatment conferred extended therapeutic protection from SARS-CoV-2-associated pathology in mice.

**Fig. 3.**
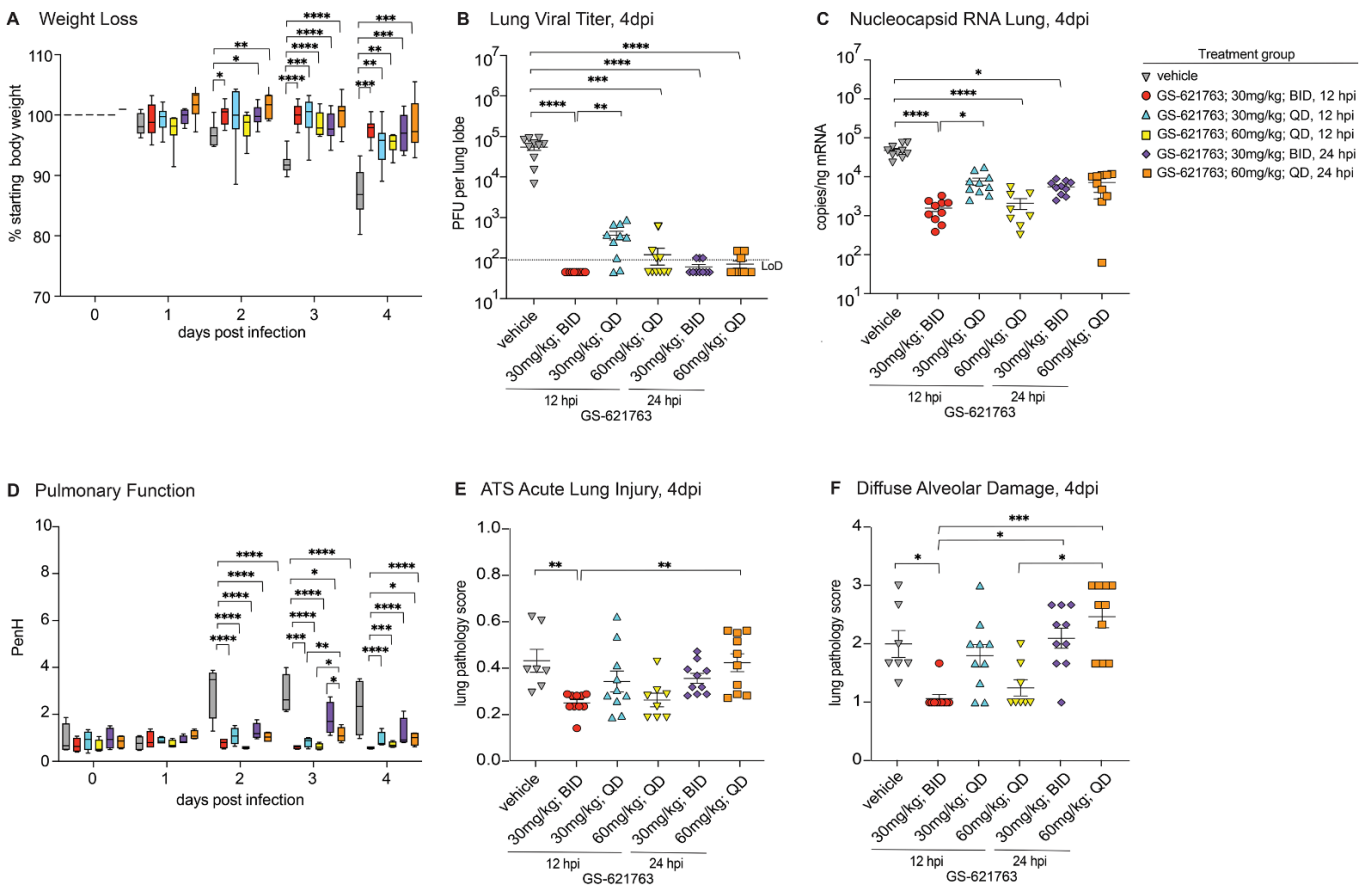
Therapeutic protection of mice against SARS-CoV-2 infection is conferred by oral administration of GS-621763 at 12 or 24 hpi. (**A**) Depicted is the percent starting weight in in mice treated therapeutically with either 30 mg/kg or 60 mg/kg BID or QD GS-621763 at 12 and 24 hpi (n=10 for all treatment groups, except n=8 for 60 mg/kg, 12 hpi QD treatment group). All mice were infected with 1x10^4^ PFU SARS-CoV-2 MA10. (**B**) Lung viral titers were measured in mice that were therapeutically treated with either 30 mg/kg or 60 mg/kg BID or QD GS-621763 at 12 and 24 hpi (n=10 for all treatment groups, except n=8 for 60 mg/kg, 12 hpi QD treatment group). All mice were infected with 1x10^4^ PFU SARS-CoV-2 MA10. The dashed line indicates the limit of detection (LoD). (**C**) Viral nucleocapsid RNA was measured in mice therapeutically treated with either 30 mg/kg or 60 mg/kg BID or QD GS-621763 at 12 and 24 hpi (n=10 for all treatment groups, except n=8 for 60 mg/kg, 12 hpi QD treatment group). All mice were infected with 1x10^4^ PFU SARS-CoV-2 MA10. (**D**) Pulmonary function was measured in mice therapeutically treated with either 30 mg/kg or 60 mg/kg BID or QD GS-621763 at 12 and 24 hpi (n=4 for all treatment groups). All mice were infected with 1x10^4^ PFU SARS-CoV-2 MA10. PenH is a surrogate measure for bronchoconstriction. (**E and F**) Lung pathology was quantified in mice therapeutically treated with either 30 mg/kg or 60 mg/kg BID or QD GS-621763 at 12 and 24 hpi (n=10 for all treatment groups, except n=8 for 60 mg/kg, 12 hpi QD treatment group, and n=7 for vehicle group). ATS acute lung injury (E) and diffuse alveolar damage (F) were scored in a blinded manner. Data were analyzed using two-way ANOVA (weight loss and lung function) and Kruskal-Wallis test (lung titer, lung viral RNA, and pathology scores), *p<0.05, **p<0.005, ***p<0.0005, ****p<0.0001. Box and whisker plots in (A) and (D) show Minimum (Min) to Maximum (Max) values. Data in (B), (C), (E), and (F) are shown as mean ± SEM.

To determine if the potent therapeutic efficacy of GS-621763 observed with early intervention (8 hours after infection) would extend to later times post infection, we designed a therapeutic efficacy study with six arms where we varied both time of oral therapy initiation and dose in BALB/c mice infected with SARS-CoV-2 MA10 (Fig. 3). As done previously, a control group of animals received vehicle twice daily beginning at 12 hours post infection (hpi). The next three arms of the study were dedicated to the 30 mg/kg GS-621763 dose, with two of the three arms receiving twice daily dosing initiated at either the 12 hpi (“30 mg/kg; BID, 12 hpi” group) or the 24 hpi (“30 mg/kg; BID, 24 hpi” group). The third 30 mg/kg arm was designed to determine if dose frequency could be reduced to once daily (quaque die, QD) if initiated early at 12 hpi (“30 mg/kg; QD, 12 hpi” group). In the last two arms, we wanted to evaluate if an increased dose of 60 mg/kg given QD beginning at 12 hours or 24 hours (“60 mg/kg; QD, 12 hpi” and “60 mg/kg; QD, 24 hpi” groups) would improve outcomes over 30 mg/kg arms while keeping the maximal amount of drug per 24 hour period uniform among most all groups (such that per 24 hours, the total amount of drug for 60 mg/kg QD = 30 mg/kg BID). Initiation of 30 mg/kg BID therapy at either 12 or 24 hours offered significant protection from weight loss (p=0.0003 each, Fig. 3A), extending the robust therapeutic phenotype observed for this dose when initiated at 8 hpi (Fig. 2B). Interestingly, when we decreased the frequency of 30 mg/kg treatment initiated at 12 hours to once daily (“30 mg/kg; QD, 12 hpi” group), we also observed a significant prevention of body weight loss (p=0.0033, Fig. 3A); thus, concentrations of drug when administered once a day and begun early (at 12 hours) in the course of infection were sufficient to prevent disease progression. Increasing the dose to 60 mg/kg QD initiated at either 12 or 24 hours offered similar protection from weight loss observed with vehicle treatment as the 30 mg/kg groups (Fig. 3A).

As body weight loss is a crude marker of viral pathogenesis, we next measured multiple virological, physiologic, and pathologic metrics of disease. First, we measured the abundance of infectious virus in lung tissue on 4 dpi. Unlike vehicle-treated animals who harbored an average titer of 5.6x10^4^ PFU per lung lobe, all GS-621763 dose groups had significantly reduced abundance of infectious virus in lung tissue, with the average titers of most groups falling below the limit of detection (50 PFU) (p<0.0001, Fig. 3B). Interestingly, 30 mg/kg delivered QD had significantly elevated viral lung titers (mean titer = 4x10^2^ PFU, p=0.0101) as compared to its BID counterpart (mean titer ≤ 90 PFU). A similar trend among treatment groups was observed when measuring the concentration of SARS-CoV-2 subgenomic and genomic nucleocapsid (N) RNA in parallel lung tissues (Fig. 3C). All 30 mg/kg groups significantly reduced abundance of SARS-CoV-2 RNA in lung tissue as compared to vehicle-treated animals (p<0.0001). As observed for infectious titers, 30 mg/kg given QD daily beginning at 12 hpi had elevated concentrations of N RNA as compared to 30 mg/kg given BID beginning at 12 hpi, suggesting that trough or daily exposure concentrations of drug with QD dosing are insufficient to suppress replication similarly to BID dosing. Increasing the once-daily dose to 60 mg/kg to raise the daily exposure and trough concentrations offered similar reductions in SARS-CoV-2 N RNA as compared to 30 mg/kg BID when initiated at 12 hpi, but the abundance of viral RNA in the higher dose 60 mg/kg group initiated at 24 hpi were not different than vehicle (Fig. 3C). To determine if incomplete suppression of infectious virus production on 4 dpi in all three QD arms (Fig. 3B) was due to selection of drug resistant virus populations, we deep sequenced total RNA utilized for reverse transcription quantitative polymerase chain reaction (RT-qPCR, Fig. 3C) from select animals that had measurable infectious virus. We found that similar numbers of nucleotide changes in vehicle and treated animals tested (table S1). We then examined the identity and frequency of non-synonymous changes in RdRp, the target of the antiviral activity of GS-621763. Amino acid changes in RdRp occurred at low frequency in all animals assessed (Cut off = 2%, Median = 3.1, Min = 2.1, Max = 10.7) (table S2). Similar mutational patterns were shared by all groups evaluated and known RDV resistance mutations were not detected (fig. S4) (*33*, *34*). These data suggest that the incomplete suppression of virus replication in animals receiving GS-621763 once daily was driven by insufficient concentrations of drug rather than acquisition of antiviral resistance. Although vehicle-treated animals exhibited substantial loss of pulmonary function as measured by WBP, this was largely prevented with GS-621763 therapy (Fig. 3D). All therapy groups initiated at 12 hpi were equally protected from loss of pulmonary function as measured by the PenH metric (Fig. 3D). Of all groups actively receiving GS-621763, animals in the 30 mg/kg BID, 24 hpi group had a measurable loss of lung function on 3 dpi that resolved by 4 dpi, but this phenotype was not observed in other groups. We then scored lung tissue sections for the histologic features of acute lung injury and alveolar damage. Only 30 mg/kg BID initiated at 12 hpi significantly reduced ALI scores as compared to those in vehicle-treated animals (p=0.0088, Fig. 3E). In addition, the 30 mg/kg BID, 12 hpi group had significantly lower ALI scores as compared to the 60 mg/kg QD, 24 hpi group (p=0.0117, Fig. 3E). Only 30 mg/kg given twice a day at 12 hpi most dramatically reduced DAD scores, but this protection from lung pathology was lost if given once per day or if initiated at 24 hpi (Fig. 3F). The high dose of 60 mg/kg QD when initiated at 12 hpi improved DAD scores as compared to similarly treated animals that began treatment at 24 hpi (Fig. 3F). Collectively, these data demonstrate that GS-621763 therapy can improve both virologic and pathogenic metrics, but the degree of improvement was dependent on time of initiation and dose frequency.

### The therapeutic efficacy of GS-621763 is similar to MPV.

**Fig. 4.**
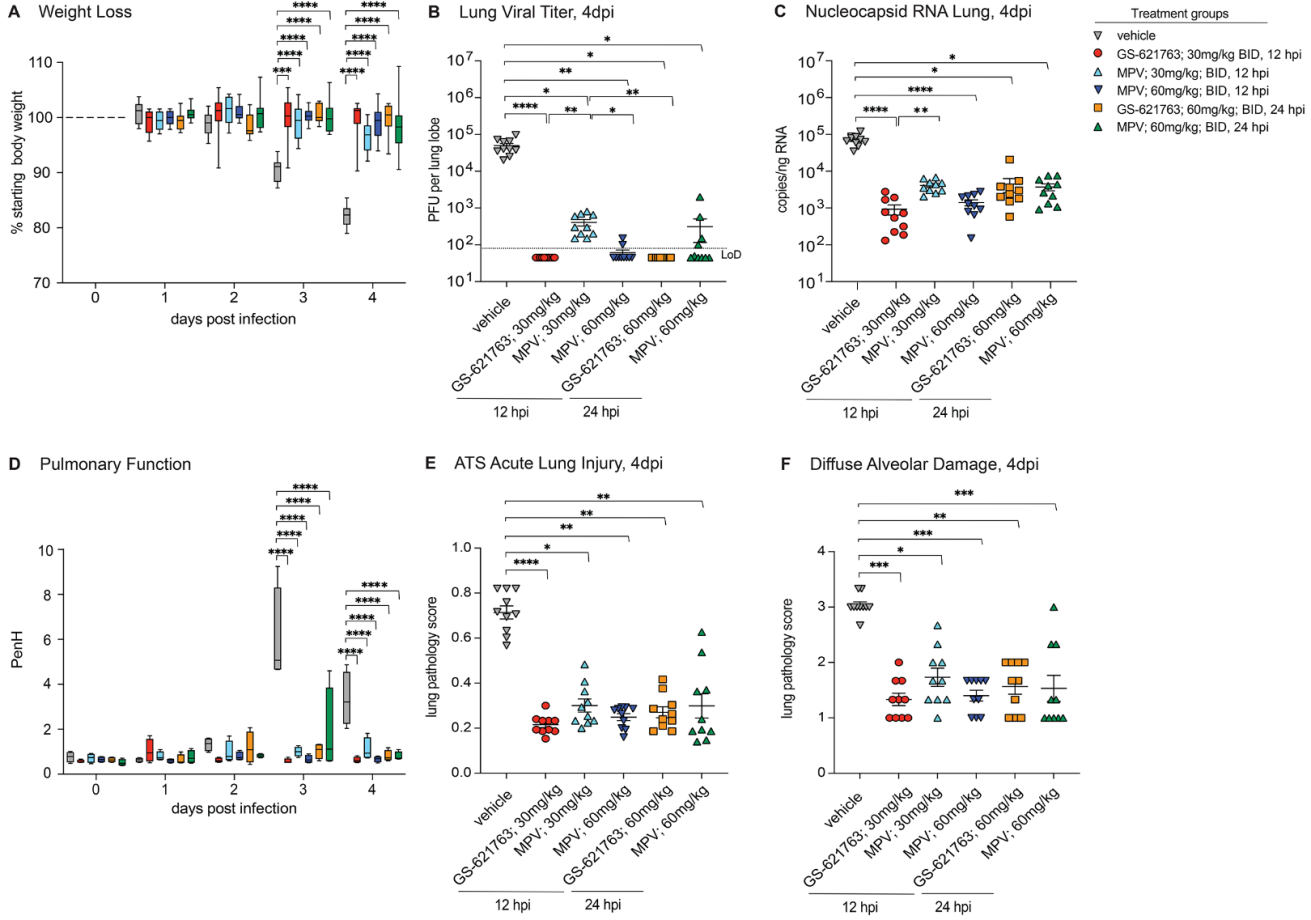
Evaluation of therapeutic intervention of GS-621763 in comparison to MPV. (**A**) Depicted is the percent starting weight in in mice treated therapeutically with either 30 mg/kg or 60 mg/kg GS-621763 or MPV at 12 or 24 hpi (n=10 for all treatment groups). All mice were infected with 1x10^4^ PFU SARS-CoV-2 MA10. (**B**) Lung viral titers were measured in mice that were therapeutically treated with either 30 mg/kg or 60 mg/kg GS-621763 or MPV at 12 or 24 hpi (n=10 for all treatment groups). All mice were infected with 1x10^4^ PFU SARS-CoV-2 MA10. The dashed line indicates the limit of detection (LoD). (**C**) Viral Nucleocapsid RNA was measured in mice therapeutically treated with either 30 mg/kg or 60 mg/kg GS-621763 or MPV at 12 or 24 hpi (n=10 for all treatment groups). All mice were infected with 1x10^4^ PFU SARS-CoV-2 MA10. (**D**) Pulmonary function was measured in mice therapeutically treated with either 30 mg/kg or 60 mg/kg GS-621763 or MPV at 12 or 24 hpi (n=4 for all treatment groups). All mice were infected with 1x10^4^ PFU SARS-CoV-2 MA10. PenH is a surrogate measure for bronchoconstriction. (**E and F**) Lung pathology was quantified in mice therapeutically treated with either 30 mg/kg or 60 mg/kg GS-621763 or MPV at 12 or 24 hours (n=10 for all treatment groups). All mice were infected with 1x10^4^ PFU SARS-CoV-2 MA10. ATS acute lung injury (E) and diffuse alveolar damage (F) were scored in a blinded manner. Data were analyzed using two-way ANOVA (weight loss and lung function) and Kruskal-Wallis test (lung titer, lung viral RNA, and pathology scores), *p<0.05, **p<0.005, ***p<0.0005, ****p<0.0001. Box and whisker plots in (A) and (D) show Minimum (Min) to Maximum (Max) values. Data in (B), (C), (E), and (F) are shown as mean ± SEM.

MPV is an oral nucleoside analog prodrug antiviral which has recently received EUA approval with demonstrated antiviral efficacy in mice against several emerging CoV including SARS-CoV, MERS-CoV, and SARS-CoV-2 (*19*, *20*, *22*, *23*). Like GS-621763, MPV is a prodrug which is metabolized in vivo into a parental nucleoside (β-D-N4-hydroxycytidine, NHC) in its metabolic progression toward the antiviral active triphosphate (*21*). To determine if GS-621763 would provide similar protection as MPV, we then designed comparative therapeutic efficacy studies in the mouse model of SARS-CoV-2 pathogenesis described above. Pre-efficacy pharmacokinetic studies in BALB/c mice (30 mg/kg or 100 mg/kg) were performed with MPV and showed dose proportional increases in NHC plasma exposures (fig. S5). Pharmacokinetic modeling then determined that a daily 120 mg/kg dose (given 60 mg/kg BID) would result in exposures similar to that observed in humans receiving 800 mg BID, a dose being evaluated in a human clinical trial (*23*). The comparative efficacy study included a vehicle group and 5 additional groups receiving two doses of MPV or GS-621763 per day 12 hours apart (BID). Three arms of the study began dosing at 12 hpi: 30 mg/kg GS-621763, 30 mg/kg MPV (0.5× human equivalent dose) or 60 mg/kg MPV (1× human equivalent dose). At 24 hpi, we began dosing of two additional groups: 60 mg/kg GS-621763 or 60 mg/kg MPV. Whereas SARS-CoV-2 MA10 infection caused rapid weight loss in vehicle control animals, all animals receiving either GS-621763 or MPV beginning at either time point were protected from weight loss (Fig. 4A). Similarly, vehicle-treated animals had expectedly high concentrations of infectious virus which was significantly reduced in all treatment groups, independent of drug type or initiation time (p<0.0001 for all, Fig. 4B). When treatment was initiated at 12 hpi, 30 mg/kg GS-621763 and 60 mg/kg MPV reduced concentrations of infectious virus below the limit of detection, whereas low but measurable titers of virus were uniformly observed in the 30 mg/kg MPV (Fig. 4B). To understand the relationship between abundance of infectious virus and viral RNA in lung tissue, we performed a SARS-CoV-2 RT-qPCR assay on parallel tissues utilized for plaque assay. The trends observed with infectious virus was mirrored in the RT-qPCR data. All groups receiving antiviral therapy had significantly reduced abundance of viral RNA (p<0.0001, p=0.0157, p<0.0001, and p=0.0410, respectively), and the difference noted among 30 mg/kg GS-621763 and 30 mg/kg MPV infectious virus titers was paralleled in these data (Fig. 4C). Similar to weight loss data, vehicle-treated animals had a significant loss of pulmonary function as measured by WBP on both 3 and 4 dpi (p<0.0001 for all), which was prevented in all groups receiving antiviral treatment (Fig. 4D). We then blindly evaluated lung tissue sections for the pathological manifestations of ALI and DAD using the two complementary histologic tools described above. Congruent with the above data, ALI scores in all antiviral therapy groups were significantly reduced as compared to vehicle controls (p<0.0001, p=0.005, p=0.0491, p=0.0014, and p=0.0022, respectively, Fig. 4E). In agreement with ALI scores, the DAD histologic scores were similarly reduced in all antiviral therapy treated groups as compared to those treated with vehicle (Fig. 4F). Together, these data show that antiviral therapy with GS-621763 and MPV when initiated early or at the peak of virus replication can both diminish virus replication and improve disease outcomes.

## DISCUSSION

Three human CoVs have emerged in the past 20 years, first with SARS-CoV in 2002 to2003, then MERS-CoV in 2012, and most recently, SARS-CoV-2 in 2019 (*1*, *2*, *35*). Vaccine availability, vaccine hesitancy, and the ongoing evolution and emergence of VOCs are collectively delaying the global control of pandemics and potential achievement of global population-level immunity (*16*, *36*–*38*). As such, there is an acute need for broad-spectrum antivirals to treat COVID-19. In addition, due to the emergence potential of the CoV family, we must also actively develop broadly acting therapies to prepare for those that may emerge in the future. Both RDV and MPV are examples of nucleoside analogs with broad activity against genetically diverse CoV (*39*). Here, we show that GS-621763, a prodrug that improves the oral delivery of parent nucleoside GS-441524, is yet another example of an orally bioavailable nucleoside analog prodrug that is effective against SARS-CoV-2 and a genetically unrelated emerging CoV, MERS-CoV.

Orally available and broadly acting antiviral therapies that target conserved viral proteins with high resistance barrier will maximize therapeutic utility against future emerging CoV. SARS-CoV-2 has undergone a considerable amount of genetic evolution since its emergence in 2019; each time a globally dominant SARS-CoV-2 VOC has emerged, it has been replaced by a new VOC harboring more concerning characteristics such as replicative capacity or transmissibility (*40*). The majority of genetic changes have been localized to viral proteins decorating the surface of the virus particle like the viral spike protein, which has mutated to eradicate epitopes targeted by early and promising monoclonal antiviral therapies, rendering them less active against newer VOCs (*41*). Antivirals like RDV, MPV, and PF-07321332 (Paxlovid) target highly conserved enzymes required for virus replication that have remained relatively invariant as new VOCs arise (*42*). In fact, only one predominant and defining mutation, P323L (Nsp12), has been observed and sustained in the RdRp of multiple VOCs including beta, gamma, delta, and omicron; this residue does not reside in known functional domains expected to interfere with nucleoside analog drugs that target RdRp (*43*). Importantly, Cox *et al*. has recently reported that GS-621763 was potently antiviral against alpha, beta, and gamma SARS-CoV-2 VOCs (*32*). The widespread use of both RDV and mAb therapies in the United States and globally has been limited by the necessity of delivery by intravenous infusion, which in turn requires access to qualified health care staff and facilities. Thus, the recent FDA EUA-approval of two oral antiviral therapies available to certain outpatient populations, MPV and Paxlovid, are likely to positively impact public health by shortening the disease duration and by preventing the hospitalization of high-risk individuals, although cost and access will ultimately guide their global utility. In addition, oral therapies used alone and in combination are needed to minimize the potential for resistance that could arise with monotherapy, as has been observed for the influenza therapies baloxavir and oseltamivir (*44*). Thus, there is a continued and global need for the development of antivirals to treat COVID-19 and infection with future emerging CoVs.

In this study, we utilized a mouse adapted SARS-CoV-2 variant, SARS-CoV-2 MA10, in wild-type BALB/c mice (*18*, *31*). Mice infected with this virus develop severe lung disease reminiscent of that seen with severe COVID-19, including the development of ALI and respiratory failure (*31*). It is important to note that the disease resulting from SARS-CoV-2 MA10 infection in mice is compressed as compared to that observed in humans with virus titers peaking in the mouse lung between 24 and 48 hours after infection, rapid loss of pulmonary function beginning 2 to 3 dpi, rapid weight loss within the first 4 days of infection, lung pathology consistent with ALI peaking 4 to 6 dpi, and virus-induced mortality within a week of infection. This is markedly different than COVID-19 in humans, where virus titers peak in the upper airway within the first week, but viral RNA shedding can be observed for as long as 24 days and symptoms can take weeks to months to resolve (*45*–*47*). Because of this caveat associated with our mouse model, the time in which to intervene with a direct acting antiviral and sufficiently improve outcomes is curtailed in mice as compared to humans. Our recent study with RDV exemplifies this, where we found the degree of therapeutic benefit in mice infected with SARS-CoV-2 MA10 was dependent on the time of initiation (*18*). Here, we show that oral administration with GS-621763 prevents body weight loss, loss of pulmonary function, severe lung pathology and virus replication when administered at 12 and 24 hours after infection. Although we observed improvement in some metrics with RDV initiated at 24 hours in our prior studies (*18*), GS-621763 therapy initiated at a similar time comparatively improved all metrics assessed. Thus, we provide proof-of-concept preclinical data that the orally bioavailable ester analog of GS-441524, GS-621763, can exert potent antiviral effects in vivo during an ongoing SARS-CoV-2 infection. Lastly, we show that GS-621763 therapy provides similar degrees of protection from SARS-CoV-2 pathogenesis as MPV, an FDA-approved oral nucleoside analog prodrug effective against SARS-CoV-2 in mice. Future directions are focused on extending these studies to evaluate the efficacy of combinations of antivirals in our models of SARS-CoV-2 pathogenesis and in other models that can evaluate the blockade of transmission, such as hamsters and ferrets.

Although we demonstrate that GS-621763 exerts antiviral activity against multiple emerging CoV and in vivo therapeutic efficacy in mouse models of SARS-CoV-2, our study is not without limitations. First, the spectrum of antiviral breadth against the CoV family remains undefined. Although we evaluated antiviral activity against SARS-CoV-2 and MERS-CoV, we did not assess activity against endemic common cold causing CoV, enzootic CoV, or current SARS-CoV-2 VOCs. Second, we show successful antiviral therapy with GS-621763 in mice but further studies in non-rodent preclinical models and human testing are ultimately needed to understand how our studies translate to humans. Lastly, we did not determine the time at which GS-621763 therapy fails to improve outcomes or reduce virus replication. Future studies should further define the breadth of GS-621762 activity against the CoV family and the timing of therapeutic intervention.

In summary, we provide preclinical data demonstrating the in vitro antiviral activity and in vivo therapeutic efficacy of an orally bioavailable nucleoside analog prodrug, GS-621763. The data provided herein supports the future evaluation of orally bioavailable prodrugs of GS-441524 in humans with COVID-19. If safe and effective, this class of RdRp inhibitors could become part of the arsenal of existing oral antivirals that are desperately needed to address a global unmet need for both the ongoing COVID-19 pandemic and CoV pandemics of the future.

## MATERIALS AND METHODS

### Study design

The primary goal of this study was to determine if the orally bioavailable prodrug of GS-441524 exhibited antiviral activity against SARS-CoV-2. Using multiple in vitro models, including human primary cells, we measured the antiviral activity of GS-621763 on SARS-CoV-2 and MERS-CoV. Primary cell cultures (HAE and NHBE) were derived from cells from individual human donors. Cytotoxicity was assessed in the A549-hACE2 and Calu-3 2B4 human lung cell lines. In vitro experiments were performed in triplicate unless otherwise stated. Drug effects were measured relative to vehicle controls. The second goal was to assess therapeutic efficacy of GS621763 in mouse models of severe SARS-2-CoV disease. The in vivo efficacy studies were intended to gain the data required to justify further testing in non-human primates and collectively inform future human clinical trials. Mice were age and sex matched and randomly assigned into groups prior to infection and prior to treatment. Most conditions evaluated in therapeutic efficacy studies were repeated at least once. Pathology scoring was performed in a blinded manner.

### Small molecule drugs, synthesis and formulation

GS-621763, RDV, and GS-441524 were synthesized at Gilead Sciences Inc., and their chemical identity and purity were determined by nuclear magnetic resonance, and high-performance liquid chromatography (HPLC) analysis (*28*). GS-621763, GS-441524, RDV were made available to the University of North Carolina (UNC) at Chapel Hill under a materials transfer agreement with Gilead Sciences. MPV was purchased from MedChemExpress LLC with a purity of 95% based on HPLC analysis. Small molecules were solubilized in 100% dimethyl sulfoxide (DMSO, EMD Millipore) for in vitro studies. For in vivo efficacy studies, GS-621763 was solubilized in vehicle containing 2.5% DMSO (EMD Millipore), 10% Kolliphor HS-15 (Sigma), 10% Labrasol (Gattefosse); 2.5% Propylene glycol (Spectrum), 75% Water (final formulation pH 2) while MPV was solubilized in vehicle containing 2.5% Kolliphor RH-40, 10% Polyethylene glycol 300, and 87.5% Water.

### In vivo plasma pharmacokinetic analysis of GS-621763 and MPV

Mice were orally administered either a single dose of GS-621763 or two doses of MPV (BID, 12 hours apart) in vehicles noted above. GS-621763 was given at either 5 or 20 mg/kg and MPV at either 30 or 100 mg/kg. Plasma was serially isolated from 4 mice at 0.25, 1, 2, 8 and 24 hours post GS-621763 administration. Plasma was isolated from alternating groups of 4 mice per timepoint at 0.5, 2, 6, 12 (pre-second dose), 12.5, 18, and 24 hours post MPV administration. Twenty μL of plasma was added to a mixture containing 250 μL of methanol and 25 μL of internal standard solution and centrifuged, and 250 μL of resulting supernatant was then transferred, filtered (Agilent Captiva 96, 0.2 μm), and dried under a stream of nitrogen at 40°C. Following reconstitution in a mixture of 5% acetonitrile and 95% water, a 10 μL aliquot was injected onto an Liquid chromatography with tandem mass spectrometry (LC-MS/MS) system. Plasma concentrations of either GS-621763 and GS-441524 or MPV and N-hydroxycytidine (NHC) were determined using 8 to 10-point calibration curves spanning at least 3 orders of magnitude with quality control samples to ensure accuracy and precision, prepared in normal mouse plasma. Analytes were separated by a 50 mm × 3.0 mm, 2.55 μm Synergi Polar-RP column (Phenomenex) using a multi-stage linear gradient from 5% to 95% acetonitrile in mobile phase A at a flow rate of 1 mL/min.

### Quantitation of GS-441524 metabolites in the lung following oral GS-621763 administration in BALB/c mice

Lungs from all mice administered GS-621763 were quickly isolated at 24 hours post-dose and immediately snap frozen in liquid nitrogen. On dry ice, frozen lung samples were pulverized and weighed. Dry ice-cold extraction buffer containing 0.1% potassium hydroxide and 67 mM ethylenediamine tetraacetic acid (EDTA) in 70% methanol, containing 0.5 μM chloro-adenosine triphosphate as internal standard, was added and homogenized. After centrifugation at 20,000 × g for 20 min, supernatants were transferred and dried in a centrifuging evaporator. Dried samples were then reconstituted with 60 μL of mobile phase A, containing 3 mM ammonium formate (pH 5) with 10 mM dimethylhexylamine (DMH) in water and centrifuged at 20,000 × g for 20 min. Final supernatants transferred to HPLC injection vials. An aliquot of 10 μL was subsequently injected onto an API 6500 LC-MS/MS system for analysis of GS-441524 and its phosphorylated metabolites, performed using a similar method as described previously (*19*).

### Viruses and plaque assay

Recombinant SARS-CoV-2 MA10 virus was generated as described previously (*31*). For virus titration by plaque assay, the caudal lobe of the right lung was homogenized in phosphate-buffered saline (PBS), and the resulting homogenate was serial-diluted and inoculated onto confluent monolayers of Vero E6 cells (American Type Culture Collection, ATCC, CRL1586), followed by agarose overlay. Plaques were visualized with overlay of neutral red dye on day 3 after infection (*31*).

### In vitro assays for SARS-CoV-2 antiviral activity

Human lung epithelial cell line A549 (ATCC # CCL185) stably expressing hACE2 were plated at a density of 20,000 cells per well in 100 μL in black-walled clear-bottom 96-well plates 24 hours prior to infection (*48*). GS-621763, RDV, GS-441524, were diluted in 100% DMSO (1:3) resulting in a 1000X dose response from 10 to 0.002 mM (10 to 0.002 μM final). All conditions were performed in triplicate. In a Biosafety Level 3 Laboratory (BSL3), medium was removed, and cells were infected with 100 μL SARS-CoV-2 nLUC (multiplicity of infection (MOI) = 0.008) for 1 hour at 37°C. After this incubation, virus was removed and wells were washed (150 μL) with infection media (Dulbecco’s Modified Eagle’s Medium (DMEM), 4% fetal bovine serum (FBS), 1X antibiotic/antimycotic). Infection media (100 μL) containing a dose response of drug was then added. Plates were incubated at 37°C for 48 hours. NanoGlo assay was performed at 48 hpi. Sister plates were exposed to drug but not infected to gauge cytotoxicity using a CellTiter-Glo assay (CTG, Promega) at 48 hours post treatment.

Normal human bronchial epithelial (NHBE) cells (donor 41219) were purchased from Lonza (Cat# CC-2540) and maintained in Bronchial Epithelial Cell Growth Medium (BEGM) (Lonza, Cat# CC-3170) with all provided supplements in the BulletKit. Cells were passaged 2 to 3 times per week to maintain sub-confluent densities and are used for experiments at passages 2 to 4. NHBE cells were seeded in 24-well plates at 1x10^5^ cells in a final volume of 0.5 mL BEGM. Cultures were incubated overnight 37°C with 5% CO_2_. On the following day, media was replaced with 0.5 mL fresh Bronchial Epithelial Cell Growth Medium (BEGM). Cultures were treated with 1:3 serial dilutions of compound using an Hewlett Packard (HP) D300e digital dispenser with normalization to the highest concentration of DMSO in all wells (<1% final volume). The cells were then infected with 0.1 mL SARS-CoV-2-fLUC diluted in BEGM at an MOI of 5. Uninfected and untreated wells were included as controls to determine compound efficacy against SARS-CoV-2-fLUC. Following incubation with compound and virus for 24 hours at 37°C with 5% CO_2_, culture supernatant was removed from each well and replaced with 0.3 mL of ONE-Glo luciferase reagent (Promega). The plates were shaken at 400 rpm for 10 min at room temperature. 0.2 mL of supernatant from each well was transferred to a 96-well opaque plate (Corning) and luminescence signal was measured using an EnVision plate reader (Perkin Elmer). Values were normalized to the uninfected and infected DMSO controls (0% and 100% infection, respectively). Data was fit using a four-parameter non-linear regression analysis using GraphPad Prism. EC_50_ values were then determined as the concentration reducing the firefly luciferase signal by 50%. The compiled data were generated based on least two independent experimental replicates, each containing four technical replicates for each concentration.

### MERS-CoV Antiviral Assay

The antiviral activity of GS-621763 against MERS-CoV was determined in a human lung epithelial cell line-based assay adapted from Sheahan *et al*. (*19*). The human lung epithelial cell line, Calu-3 2B4 (2B4, obtained as a gift from Dr. Kent Tseng, University of Texas Medical Branch), was maintained in DMEM (Gibco), 20% FBS (Hyclone), and 1X antibiotic/antimycotic (Gibco) (*49*). Twenty-four hours after plating 5x10^4^ cells per well, fresh medium was added. In triplicate, cells were exposed to serial dilutions of compound and control compounds (GS-441524 and RDV) in “infection medium” (modified growth medium similar to that above but with 10% FBS) and immediately infected for 1 hour with MERS-nLUC at a MOI of 0.008. After 1 hour, virus was removed, cultures were rinsed once, and infection medium containing dilutions of drug or vehicle was added. DMSO (0.05%) was constant in all conditions. For cytotoxicity measures, non-infected sister plates were similarly treated at the time of infection. At 48 hpi, virus replication was measured by nLUC assay (Promega) and cytotoxicity in non-infected plates was measured using a CellTiter-Glo (Promega) assay and then read on a Promega Glomax plate reader. The EC_50_ value was defined in GraphPad Prism 9.0 (GraphPad) as the concentration at which there was a 50% decrease in viral replication using non-infected wells (100% inhibition) and infected and vehicle treated wells (0% inhibition) as controls. CC_50_ values were determined through comparison of data with that from cell-free (100% cytotoxic) and vehicle only (0% cytotoxic) samples.

### Mouse studies and in vivo infections

All mouse studies were performed at the University of North Carolina (Animal Welfare Assurance #A3410-01) using protocols (#20-059) approved by the University of North Carolina Institutional Animal Care and Use Committee. All animal work was approved by the Institutional Animal Care and Use Committee at University of North Carolina at Chapel Hill according to guidelines outlined by the Association for the Assessment and Accreditation of Laboratory Animal Care and the US Department of Agriculture. All work was performed with approved standard operating procedures and safety conditions for SARS-CoV-2. Our institutional BSL3 facilities are designed to conform to the safety requirements recommended by Biosafety in Microbiological and Biomedical Laboratories, the US Department of Health and Human Services, the Public Health Service, the Centers for Disease Control and Prevention, and the National Institutes of Health. Laboratory safety plans have been submitted, and the facility has been approved for use by the University of North Carolina Department of Environmental Health and Safety and the Centers for Disease Control and Prevention.

Even groups (n=10, or less as indicated) of 10-week-old female BALB/c mice (Envigo; #047) were used in all in vivo efficacy studies. For infection, mice were anesthetized with a mixture of ketamine/xylazine and infected with 10^4^ PFU of SARS-CoV-2 MA10 in 50 μl PBS intranasally. Vehicle or GS-621673 was administered orally at the dosages and timepoints as indicated. Mice were monitored daily for body weight changes and for lung function by whole-body plethysmography. At 4 dpi, mice were euthanized, and lung tissue was harvested for viral titer analysis, RNA isolation, and histology, and lung congestion scores were estimated (*31*). Samples for viral load determination and for RNA isolation were stored at −80°C until used; histology samples were inactivated in 10% neutral-buffered formalin solution (NBF) and stored at 4°C until further processing.

### Histology and lung pathology scoring

Two separate lung pathology scoring scales, Matute-Bello and Diffuse Alveolar Damage (DAD), were used to quantify acute lung injury (ALI) (*20*). For Matute-Bello scoring samples were blinded and three random fields of lung tissue were chosen and scored for the following: (A) neutrophils in alveolar space (none = 0, 1 to 5 cells = 1, greater than 5 cells = 2), (B) neutrophils in interstitial space (none = 0, 1 to 5 cells = 1, greater than 5 cells = 2), (C) hyaline membranes (none = 0, one membrane = 1, greater than 1 membrane = 2), (D) Proteinaceous debris in air spaces (none = 0, 1 instance = 1, more than 1 instance = 2), (E) alveolar septal thickening (< 2x mock thickness = 0, 2–4x mock thickness = 1, > 4x mock thickness = 2). Scores from A to E were put into the following formula score = [(20x A) + (14 x B) + (7 x C) + (7 x D) + (2 x E)]/100 to obtain a lung injury score per field and then averaged for the final score for that sample.

In a similar way, for DAD scoring, three random fields of lung tissue were scored for the in a blinded manner for: 1= absence of cellular sloughing and necrosis, 2= uncommon solitary cell sloughing and necrosis (1 to 2 foci per field), 3=multifocal (3 or more foci) cellular sloughing and necrosis with uncommon septal wall hyalinization, or 4=multifocal (greater than 75% of field) cellular sloughing and necrosis with common or prominent hyaline membranes. To obtain the final DAD score per mouse, the scores for the three fields per mouse were averaged.

### RNA isolation and RT-qPCR

Lung tissue from SARS-CoV-2 infected mice was homogenized using glass beads in TRIzol Reagent (Invitrogen). Equal volume of 100% ethanol was mixed with the TRIzol homogenate and processed using Direct-Zol RNA MiniPrep Kit (Zymo) to extract viral RNA. Optional DNase I treatment was conducted to ensure the adequate removal of unwanted DNA. Eluted RNA was coupled with TaqMan Fast Virus 1-step Master Mix (Applied Biosystems) and nCOV_N1 primers/probe (IDT) to quantify viral load by RT-qPCR. Samples were plated on a MicroAmp EnduraPlate (Applied Biosystems) and run using a QuantStudio 6 Real-Time PCR System (Applied Biosystems) to obtain viral titers. The following PCR program was run: 50°C for 5 min, 95°C for 20 s, followed by 45 cycles of 95°C for 3 s and 60°C for 30 s. The sequences of the 2019-nCOV_N1 primers and probe were as follows: Forward primer: GAC CCC AAA ATC AGC GAA AT, Reverse primer: TCT GGT TAC TGC CAG TTG AAT CTG, Probe: AC CCC GCA TT ACG TTT GGT GGA CC (CDC N1 qRT-PCR assay (*50*). SARS-CoV-2 standard curve RNA was produced by PCR amplification of SARS-CoV-2 nucleocapsid in which a 5′ T7 polymerase promoter was introduced. This amplicon was used as template to generate in vitro transcribed RNA which was then quantified and serially diluted (108 to 101 copies per μL).

### RNA sequencing

Purified RNA was prepared for sequencing using the Illumina RNA Prep with Enrichment (Illumina, 20040536) workflow, following the instructions for the Respiratory Virus Oligo Panel (Illumina, 20044311). The resulting libraries were run on a MiSeq instrument, with at least 1.5 million reads per sample. Reads were aligned to the SARS-CoV-2 MA10 genome using STAR (v2.7.9). Output BAM files were input into Geneious Prime, and variant calling was performed with a minimum of 100 reads per base and a quality score of 25, and a cutoff of 0.02 frequency variance. Variants in the nsp12 gene were averaged, then graphed along the length of the gene using a custom R script.

### Statistical Analysis

Raw, individual-level data are presented in data file S1. A two-way analysis of variance (ANOVA) followed by Tukey’s multiple comparisons test to control for false discovery rate or a one-way ANOVA (Kruskal Wallis Test) followed by Dunn’s multiple comparisons to adjust for false discovery rate were used for mouse and for in vitro experiments. All statistical calculations were performed in GraphPad Prism 9.

## Supplementary Materials

### Other Supplementary Material for this manuscript includes the following:

MDAR Reproducibility Checklist
