# Supplementary material for: Therapeutic treatment with an oral prodrug of the remdesivir parental nucleoside is protective against SARS-CoV-2 pathogenesis in mice
Source: Sci Transl Med. 2022 Mar 22:eabm3410. doi: 10.1126/scitranslmed.abm3410 (PMC8995034; doi:10.1126/scitranslmed.abm3410)
Supplement: Supplementary file 1 — Fig. S1 to S5 Tables S1 and S2 [file scitranslmed.abm3410_sm.pdf]

Science Translational Medicine
AAAS

Supplementary Materials for

Therapeutic treatment with an oral prodrug of the remdesivir parental nucleoside is protective against SARS-CoV-2 pathogenesis in mice

Alexandra Schäfer *et al.*

Corresponding author: Timothy P. Sheahan, sheahan@email.unc.edu

DOI: 10.1126/scitranslmed.abm3410

The PDF file includes:

Figs. S1 to S5
Tables S1 and S2

Other Supplementary Material for this manuscript includes the following:

MDAR Reproducibility Checklist
Data file S1

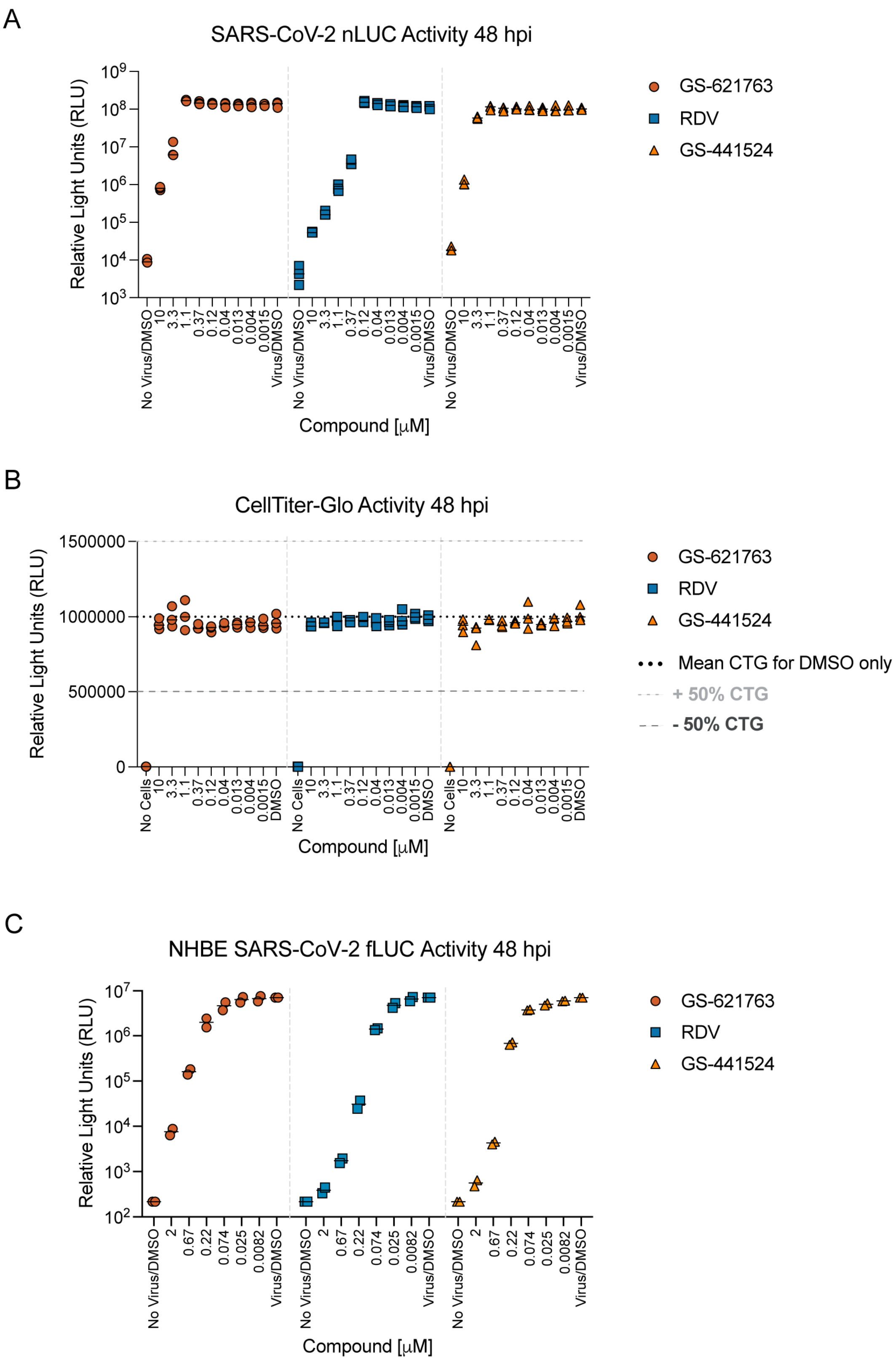

Fig. S1. In vitro potency and cytotoxicity of GS-621763, Remdesivir (RDV), and GS-441524 in A549 cells expressing human angiotensin converting enzyme 2

(A549-hACE2 cells). (**A**) Raw data are shown for the inhibition of SARS-CoV-2 replication by GS-621763, RDV, and GS-441521 in A459-hACE2 cells measured through quantitation of SARS-CoV-2 expressed nano luciferase (nLUC); data were analyzed in triplicate. (**B**) Raw data are shown for cytotoxicity in A459-hACE2 cells treated with GS-621763, RDV, and GS-441521. Cytotoxicity was measured using a CellTiter-Glo (CTG) assay and was measured in triplicates. (**C**) Raw data are shown for the inhibition of SARS-CoV-2-fLUC replication by GS-621763, RDV, and GS-441521 in normal human bronchial epithelial cultures measured through quantitation of SARS-CoV-2 expressed firefly luciferase (fLUC); data were measured in duplicates and the experiment was repeated twice.

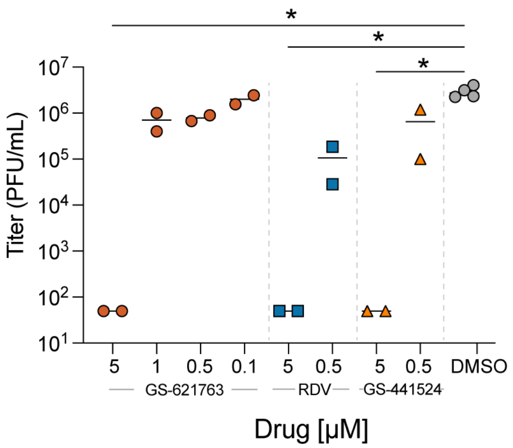

Fig. S2. GS621763 treatment reduces viral titers in primary human airway epithelial cell cultures.

Human airway epithelial cell cultures were treated with different doses of GS-621763, GS-441524, RDV, or DMSO and then infected with SARS-CoV-2 at an MOI of 0.5. Treatments were administered in duplicate. After 2 hours of infection, input virus was removed, cultures were washed once, and infectious virus titers in plaque forming units (PFU) was measured in apical washes after 72 hours. Each symbol represents the virus titer in a duplicate culture. Horizontal bars indicate median values. * indicates $p<0.05$ as determined by Kruskal-Wallis test with Dunn’s post test.

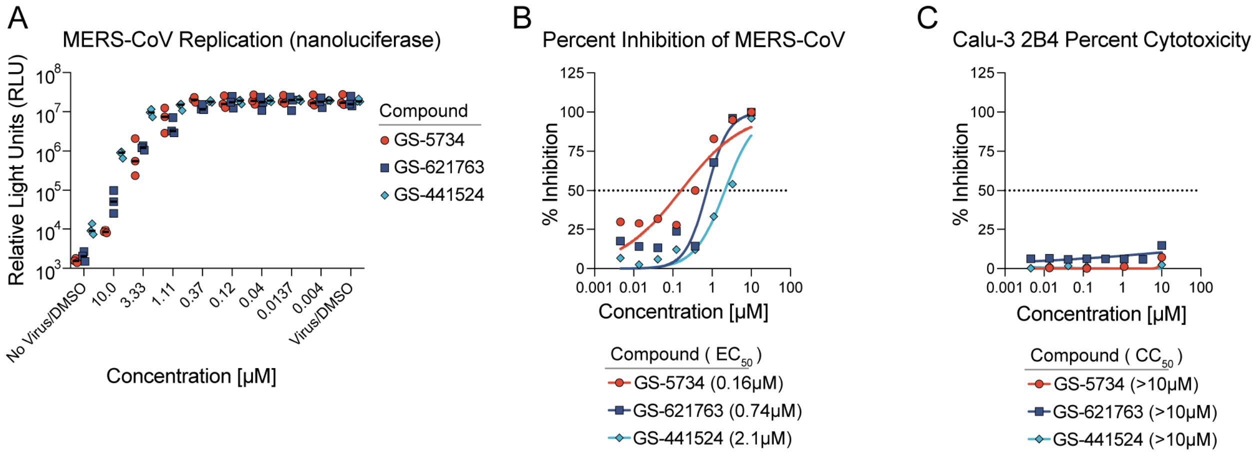

Fig. S3. Antiviral activity of GS-621763 against Middle East Respiratory Syndrome CoV (MERS-CoV).

Calu-3 2B4 cells were infected in triplicate with MERS-CoV-nLUC at a multiplicity of infection (MOI) of 0.008 in the presence of varying concentrations of indicated drug for 48 hours, after which replication was measured through quantitation of MERS-CoV–expressed nLUC. (**A**) MERS-CoV nanoluciferase (nLUC) activity was measured in Calu-3 2B4 cells treated with a dose response of GS-621763, GS-441524, or RDV. (**B**) Mean percent inhibition of MERS-CoV replication by RDV, GS-621763, or GS-441624 is shown. The horizontal line at 50% indicates the half-maximum effective concentration (EC_{50}). (**C**) Cytotoxicity was measured in non-infected Calu-3 2B4 cells treated similarly to that in (A). Viability was measured using a CellTiter-Glo assay. The horizontal line at 50% indicates the 50% cytotoxic concentration (CC_{50}). Data shown are representative of two independent experiments.

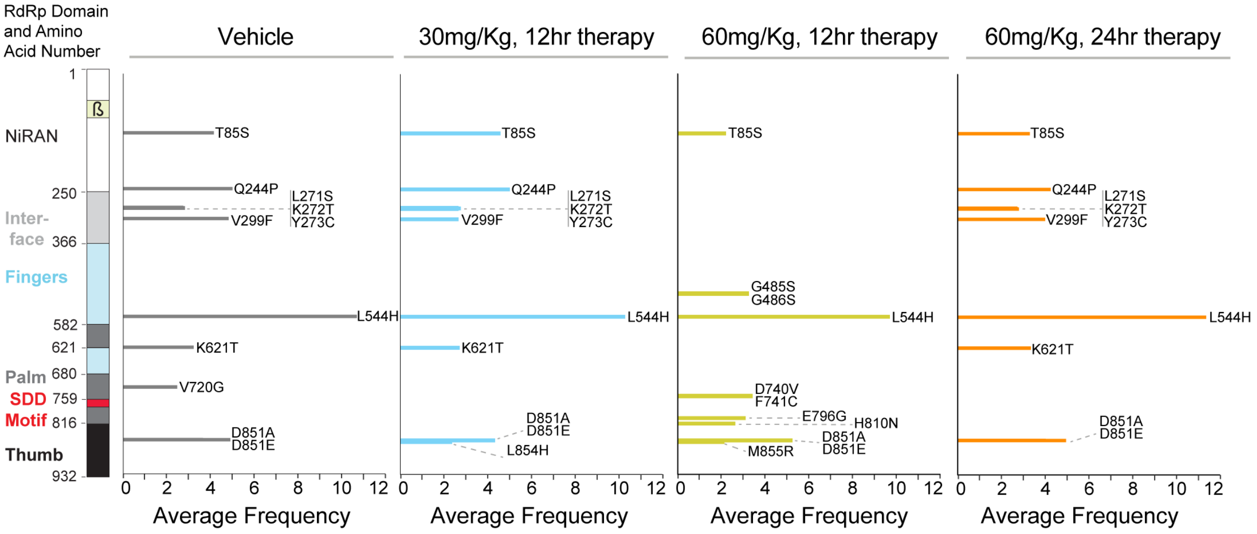

Fig. S4. Identity and frequency of non-synonymous changes in the SARS-CoV-2 RNA-dependent RNA polymerase (RdRp).

Deep sequencing is shown for total RNA utilized for reverse transcription quantitative polymerase chain reaction (Fig. 3C) from animals in the 30 mg/kg or 60 mg/kg quaque die (QD) GS-621763 treatment groups at 12 and 24 hours post-infection (n=3 or 4, respectively, for each sequenced group). The mutation locations are plotted onto the linear amino acid sequence of RdRp (y-axis). Major RdRp functional domains are noted including: nidovirus RdRp-associated nucleotidyltransferase (NiRAN), the N-terminal beta hairpin (ß), the interface domain connecting NiRAN to the main polymerase domain comprised of subdomains fingers, palm and thumb. The serine, aspartic acid, aspartic acid (SDD) catalytic site motif of the polymerase is noted. Sequencing was done to determine the identity and frequency of non-synonymous changes in RdRp. All mice were infected with $1x10^4$ PFU SARS-CoV-2 MA10.

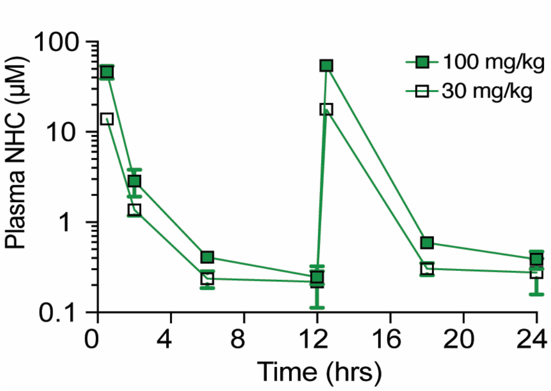

Fig. S5. Molnupiravir mouse plasma pharmacokinetics.
Plasma pharmacokinetics of N-hydroxycytidine (NHC) in uninfected BALB/c mice are shown following daily oral administration of molnupiravir at either 60 or 200 mg/kg (as either 30 or 100 mg/kg/dose given BID). Data are presented as mean±SD.

Table S1. Number of nucleotide changes in the SARS-CoV-2 genome in lung samples isolated from animals with infectious virus on 4 dpi.

Group	Animal ID	# Nucleotide Changes
**Vehicle**	1-2	152
**Vehicle**	1-4	199
**Vehicle**	2-1	184
**Vehicle**	2-4	195
**30mg/kg, QD 12hr**	5-1	172
**30mg/kg, QD 12hr**	5-3	154
**30mg/kg, QD 12hr**	5-4	204
**60mg/kg, QD12hr**	7-2	181
**60mg/kg, QD 12hr**	7-5	182
**60mg/kg, QD 12hr**	8-3	289
**60mg/kg, QD 24hr**	11-2	157
**60mg/kg, QD 24hr**	11-4	164
**60mg/kg, QD 24hr**	12-3	148
**60mg/kg, QD 24hr**	12-4	116

Table S2. Number of non-synonymous changes in the RNA-dependent RNA polymerase in animals with infectious virus titers on 4dpi.

		**Vehicle**				**30 mg/kg; QD, 12hpi**			
**Position**	**Codon**	Change	Codon Change	A.A.	Avg	Change	Codon Change	A.A.	Avg
13693	85	A -> T	ACA -> TCA	T -> S	4.2	A -> T	ACA -> TCA	T -> S	4.6
13711	91								
13761	107								
14111	224	A -> C	CAA -> CCA	Q -> P	5.0	A -> C	CAA -> CCA	Q -> P	5.0
14252	271	T -> C	UUA -> UCA	L -> S	2.8	T -> C	UUA -> UCA	L -> S	2.7
14255	272	A -> C	AAA -> ACA	K -> T	2.8	A -> C	AAA -> ACA	K -> T	2.8
14258	273	A -> G	UAU -> UGU	Y -> C	2.8	A -> G	UAU -> UGU	Y -> C	2.7
14335	299	G -> T	GUU -> TUU	V -> F	4.8	G -> T	GUU -> TUU	V -> F	2.7
14893	485								
14896	486								
15071	544	T -> A	CUU -> CAU	L -> H	10.7	T -> A	CUU -> CAU	L -> H	10.3
15302	621	A -> C	AAA -> ACA	K -> T	3.2	A -> C	AAA -> ACA	K -> T	2.7
15599	720	T -> G	GUC -> GGC	V -> G	2.5				
15659	740								
15662	741								
15827	796								
15868	810								
15992	851	AT -> CC	GAU -> GCC	D -> A	3.7	AT -> CC	GAU -> GCC	D -> A	3.4
15993	851	T -> A	GAU -> GAA	D -> E	4.9	T -> A	GAU -> GAA	D -> E	4.3
16001	854					T -> A	CUU -> CAU	L -> H	2.4
16004	855					T -> G	AUG -> AGG	M -> R	2.4
16164	908								

		60mg/kg; QD, 12pi				60mg/kg; QD, 24hpi			
**Position**	**Codon**	Change	Codon Change	A.A.	Avg	Change	Codon Change	A.A.	Avg
13693	85	A -> T	ACA -> TCA	T -> S	2.2	A -> T	ACA -> TCA	T -> S	2.675
13711	91	A -> T			2.1				
13761	107	C -> T	GAC -> GAT		2.5				
14111	224					A -> C	CAA -> CCA	Q -> P	3.625
14252	271					T -> C	UUA -> UCA	L -> S	2.1
14255	272					A -> C	AAA -> ACA	K -> T	2.125
14258	273					A -> G	UAU -> UGU	Y -> C	2.175
14335	299					G -> T	GUU -> TUU	V -> F	3.375
14893	485	G -> A	GGU -> AGU	G -> S	3.1				
14896	486	G -> A	GGC -> AGC	G -> S	3.3				
15071	544	T -> A	CUU -> CAU	L -> H	9.7	T -> A	CUU -> CAU	L -> H	10.725
15302	621					A -> C	AAA -> ACA	K -> T	2.725
15599	720								
15659	740	A -> T	GAC -> GTC	D -> V	2.4				
15662	741	T -> G	UUU -> UGU	F -> C	3.4				
15827	796	A -> G	GAA -> GGA	E -> G	3.1				
15868	810	C -> A	CAU -> AAU	H -> N	2.6				
15992	851	AT -> CC	GAU -> GCC	D -> A	3.6	AT -> CC	GAU -> GCC	D -> A	3.4
15993	851	T -> A	GAU -> GAA	D -> E	5.2	T -> A	GAU -> GAA	D -> E	4.325
16001	854								
16004	855	T -> G	AUG -> AGG	M -> R	2.1				
16164	908	T -> A	ACU -> ACA		2.9				
